# Homologous recombination is unlikely to play a major role in influenza B virus evolution

**DOI:** 10.1186/1743-422X-5-65

**Published:** 2008-05-27

**Authors:** Guan-Zhu Han, Xi-Ping Liu, Si-Shen Li

**Affiliations:** 1 National Key Laboratory of Crop Biology, College of Agronomy, Shandong Agricultural University, Tai'an 271018, China; 2 College of Life Science, Shandong Normal University, Jinan 250014, China

## Abstract

Influenza B viruses cause a significant amount of morbidity and mortality. The occurrence of homologous recombination in influenza viruses is controversial. To determine the extent of homologous recombination in influenza B viruses, recombination analyses of 2,650 sequences representing all eight segments of the influenza B viruses were carried out. Only four sequences were indentified as putative recombinants, which were verified using phylogenetic methods. However, the mosaics detected here were much likely to represent cases of laboratory-generated artificial recombinants. As in other myxoviruses, it is unlikely that homologous recombination plays a major role in influenza B virus evolution.

## Background

Influenza B viruses cause substantial morbidity and mortality in humans. As a member of the *Orthomyxoviridae *family, influenza B virus possesses a single-stranded and segmented genome of negative sense. Unlike influenza A viruses, no antigenic shift has ever been detected in influenza B viruses. No subtype divisions of surface antigens exist and two lineages, Victoria lineage and Yamagata lineage, have diverged since 1983 as defined by the phylogenetic relationship of the hemagglutinin (HA) gene [1]. All 11 genes of influenza B viruses have diverged into two new lineages prior to 1987 [2,3]. Reassortment occurs frequently among influenza B viruses and likely allows unrestricted lineage mixing [2].

To date, there is also ample evidence that various forms of non-homologous recombination, albeit rarely, occurs in influenza viruses [4-6]. However, the occurrence of homologous recombination in influenza viruses is controversial and far from proven. For influenza A viruses, Gibbs *et al. *proposed that homologous recombination had occurred in the HA gene of 1918 Spanish flu virus [7]. However, the apparent recombination event described by Gibbs *et al. *is much likely to result from a difference in the substitution rate of evolution between HA1 and HA2 [8]. More recently, Boni *et al. *demonstrate that homologous recombination is very rare or absent in human influenza A viruses through analyzing a data set of 13,852 sequences representing all eight RNA segments and of both major circulating subtypes, H3N2 and H1N1 [9]. Therefore, whether homologous RNA recombination occurs in influenza viruses is one of the key research questions in influenza virus evolution [10].

## Results and Discussion

To access whether homologous RNA recombination plays a role in the evolution of influenza B viruses, we compiled a data set of 2,650 sequences (PB2, 224; PB1, 230; PA, 230; HA, 330; NP, 236; NA, 687; MP, 332; NS, 381) representing all eight RNA segments. The sequences were obtained from the Influenza Virus Resource [11] and then aligned using Clustal X [12]. To gain an initial insight into possible recombination events, each of the eight data sets was analyzed respectively using the 3SEQ [13], the Chimaera [14], and the RDP [15] methods, which are available in RDP (Recombination detection program) software. Interestingly, all these three methods implemented got the same results. Only four potential recombinants were primarily identified (Table 1). The recombinants were distributed over only three (PB2, NA, and HA) of the eight influenza B virus RNA segments.

**Table 1:** Influenza B virus strains with significant recombination signal

Segment	Recombinant	Accession No.	Putative Parents	3SEQ p-value	Breakpoint	Δ c-AIC
PB2	B/Memphis/5/93	AY582061	B/Shiga/T30/98	4.496 × 10^-21^	1665	105.592
			B/Alaska/03/1992			
PB2	B/Norway/1/84	AF101984	B/Guangdong/05/94	6.654 × 10^-13^	1206	78.8114
			B/Chile/3162/2002			
HA	B/Memphis/5/93	AF129902	B/Houston/B56/1997	8.988 × 10^-11^	885	56.3597
			B/Houston/1/92			
NA	B/Memphis/3/93	AF129915	B/Alaska/03/1992	1.665 × 10^-14^	808	70.3841
			B/Memphis/10/97			

Recombination events were further confirmed and the exact breakpoints were identified using GARD (Genetic Algorithm Recombination Detection) online [16,17]. To better evaluate the evidence for these recombination events, the breakpoints identified by GARD were used to divide the alignment into two parts to construct phylogenetic trees respectively. Phylogenetic trees were generated using the Maximum Composite Likelihood (MCL) method for estimating evolutionary distances and neighbor-joining (NJ) method [18] in MEGA4.0 [19]. The phylogenetic trees were tested with bootstrap of 1000 replicates. The occurrence of incongruent phylogenetic trees, the most compelling evolutionary evidence for recombination, was observed for all the four putative recombinants which further confirmed the results of the recombination analyses above. Meanwhile, topological shifts for each of the recombinants have strong bootstrap support (data not shown).

However, large influenza viral genes in the databases may actually represent assembled artifactual contigs from different but homologous gene segments present in a mixed sample to begin with. Such artifactual contigs are also likely to be produced in a mixed sample by template switching during PCR amplification [20] even if only a single primer set is used. Mixture of viruses was present leading to the illusion of a recombination event as a consequence of the sequencing methodology being employed. A plausible explanation for the "recombinants" detected here is contamination by influenza virus derived PCR products, which could combine during PCR amplification to generate apparent, but artifactual recombinants. None of the three putative recombinant viruses were derived by plaque purification. Furthermore, the same laboratory was the source for all four recombinants and the one putative parental strain. As suggested in influenza A virus  [9], further work would be needed to conclusively demonstrate homologous recombination in influenza B viruses. Recombinant influenza virus must either be plaque purified or multiple clones must be isolated and sequenced from the same individual or animal host. The presence of both the recombinant and parental genotypes should be found in the sample [21]. Alternatively, homologous recombination could be demonstrated by showing that recombinant sequences form a distinct lineage circulated among multiple identified individuals [22].

## Conclusion

To sum up, our analysis showed that homologous recombination in influenza B viruses was very rare or absent and could not confer a substantial fitness advantage. Therefore, we conclude that homologous recombination is unlikely to play a major role in influenza B virus evolution. 

## Competing interests

The authors declare that they have no competing interests.

## Authors' contributions

GZH, SSL designed the study; GZH, XPL carried out the study; GZH, SSL, XPL drafted the manuscript. All authors read and approved the final manuscript.
